# DOSAGE: Dataset for Optimized Safe Antibiotic Guidelines and Estimates

**DOI:** 10.1038/s41597-025-05761-8

**Published:** 2025-10-21

**Authors:** Ferdous Wahid Anik, Marzia Zaman, Tahmina Foyez, Khondaker A. Mamun

**Affiliations:** 1 Advanced Intelligent Multidisciplinary Systems (AIMS) Lab, Institute of Research Innovation, Incubation and Commercialization (IRIIC), United International University, Dhaka, 1212 Bangladesh; 2 CMED Health Limited, Dhaka, 1206 Bangladesh; 3 Department of Pharmacy, United International University, Dhaka, 1212 Bangladesh; 4 Department of Computer Science and Engineering, United International University, Dhaka, 1212 Bangladesh

**Keywords:** Health care economics, Quality of life

## Abstract

The DOSAGE was developed to support personalized dosing regimens and pregnancy risk factors based on patient-specific characteristics, including age, weight, and renal function for 98 generics in total. The dataset was validated by healthcare professionals through a multi-step process that involved manual review and expert corrections. Data integrity, consistency, and clinical adherence were rigorously assessed through automated checks and manual reviews. The proposed dataset aims to provide clinicians with consultation tools and standardized data that they can seamlessly integrate into their current clinical workflow, thereby improving patient outcomes and overall quality of care.

## Background & Summary

Medication appropriateness is a critical aspect of healthcare that aims to maintain patient safety through accurate drug adjustments, precise medical guidelines, and standardized practiced order entry. Efficient medication management requires accurate prescription, distribution, and supervision^1^. Antibiotics are the most commonly administered medications globally^2^ in modern medicine to treat bacterial infections and improve patient outcomes^3^.

Individualized antibiotic treatment improves therapy success and lowers the risk of negative outcomes, but it also poses some challenges in medical practice^4^. Optimizing the use of antibiotics is crucial due to the frequent modification of doses based on multiple parameters, including specific diseases and patient data such as age group, body size, allergy status, and renal function^5^. Minimizing adverse effects and reducing the burden of antimicrobial resistance through the judicious and rational use of antimicrobials can significantly ameliorate inappropriateness, which can undermine healthcare standards^6–8^. To enhance treatment outcomes and mitigate resistance in patients who responsibly administer antibiotics, it implements precise microbiological diagnostics, appropriate administration, evidence-based guidelines, and ethical stewardship^9^.

Global studies reveal that antibiotic misuse remains widespread despite the availability of clinical guidelines. Antimicrobial prescriptions are issued in outpatient settings in nearly 90% of cases^10^, and approximately half of all global prescriptions are considered inappropriate^11,12^. In a national-level study of china, inappropriate antibiotic use was observed in 91.8% of cases^13^. While clinical knowledge forms the foundation of effective prescribing, factors such as clinician workload, limited consultation time, and the lack of real-time, patient-specific tools often contribute to medication errors^14^. Additionally, incomplete prescriptions and omitted dosage details further reduce the effectiveness of therapy and increase the risk of harm^15^.

Authoritative references such as the British Pharmacopoeia (BP), European Pharmacopoeia (EP), Japanese Pharmacopoeia (JP), and United States Pharmacopeia (USP) provide standardized formulations and regulatory quality controls for medication use^16^. In parallel, a wide range of digital platforms^17–24^ offers clinically valuable information on drug indications, contraindications, and dosage recommendations designed to support expert-driven decision-making through manual interpretation. However, as clinical practice increasingly incorporates automated systems and real-time support, there is a growing need of structured data for machine executable and patient-specific decision system. In response, attempts to apply natural language processing (NLP) to extract medication rules from unstructured content have highlighted challenges related to inconsistency, ambiguity, and data completeness^25^. These technical barriers limit the effectiveness of AI-enabled prescribing systems, particularly in environments that require transparent, protocol-driven decision-making. This underscores the value of curated, machine-readable datasets capable of delivering interpretable and reproducible logic grounded in validated clinical standards.

Technological interventions such as computerized physician order entry (CPOE), clinical decision support systems (CDSS), and machine learning (ML) algorithms have shown promise in enhancing medication safety by supporting accurate transcription and flagging potential prescription errors^26–28^. Hybrid approaches that integrate rule-based protocols with predictive features have been used to detect medication errors, especially in antibiotic prescribing^29^. However, many of these systems are constrained by a lack of high-quality, structured data tailored to patient-level variables such as age, weight, renal function, and comorbid risks^30^. In parallel, emerging large language models (LLMs) demonstrate strong capabilities in processing and generating natural language from extensive unstructured clinical content. Retrieval-augmented generation (RAG) further enhances this capability by incorporating up-to-date external sources such as clinical guidelines and medical literature to improve the relevance and factual grounding of model outputs^31^. Despite these advancements, studies have identified critical limitations in LLM generated content, including nondeterministic behavior and hallucinations, leading to inaccurate information that can undermine clinical validity and patient safety^32^. These challenges are particularly serious in areas like antibiotic prescribing, where accurate dosing and following clinical guidelines are critical. Therefore, there is a clear need for structured and interpretable resources that offer reliable, guideline-based support to help ensure safe and consistent decision-making in clinical practice.

The DOSAGE dataset responds to this need by rearticulating established antibiotic dosing guidance into a structured, clinically interpretable format aligned with key patient variables. Unlike patient-level datasets that record individual histories and treatment outcomes^33^, this dataset encodes prescribing logic derived from guideline-based sources which supports reproducible and context-aware dosing decisions without reliance on region-specific data.

## Methods

Our journey began by collecting a list of 98 generics of antibiotics from the Directorate General of Health Services. (DGHS) (http://www.dgdagov.info). So, we planned to incorporate all antibiotic medications in DOSAGE from various sources by defining protocols to automatically resolve prescription drug errors. To create DOSAGE we had to go through four steps which are 1. Requirement analysis 2. Protocol define for DOSAGE protocol define for DOSAGE 3. Data Collection and 4. Source identification.

### PHASE 1: Requirement Analysis

Prescribing medication for outpatients starts with considering that a patient visits a doctor, and the prescription process involves a series of critical evaluations. The doctor must first diagnose the patient’s condition and then consider several factors, including patient data, appropriate medication and risk factors. The WHO supports systematic medication prescription underpinned by an organized and evidence-based process for quality and safety in prescriptions. These processes include description of the patient’s problem, statement of the therapeutic goals, and the choice of appropriate medication therapy^34^. For DOSAGE, such an organized methodology is the key component in the construction of a robust antibiotic medication strategy. The objective of the protocol is to address the triad concerns: diagnose the patient for choosing the correct drug, prescribe medication according to patient data and consider risk factors for selected medication — all steps that play an enormous role in minimizing errors and maximizing the potential for better outcomes.

#### Diagnosis and Medication Selection

The path to diagnosis commences with the patient’s reported symptoms and their visit to the doctor, who thoroughly reviews the patient’s medical history to identify the disease. Often, the doctor confirms the diagnosis by conducting appropriate diagnostic procedures^35^. Furthermore, the doctor may prescribe antibiotics based on the patient’s physical condition without specifying the ailment or continuously reassessing the diagnosis. However, DOSAGE creates its protocols by individualizing them based on either disease-specificity or without specifying any disease for each antibiotic.

#### Dosing Adjustments Based on Patient-Specific Parameters

Appropriate antibiotic dosing is influenced by patient-specific factors such as age, weight, and renal function due to the pharmacokinetics (PK) and pharmacodynamics (PD) of drugs. For pediatric patients, dosing regimens are stratified by age and weight groups to account for developmental differences in drug metabolism, with doses typically lower for neonates and infants due to their immature kidney function, which affects drug clearance^36^. Similarly, geriatric dosing is adjusted to account for reduced renal function, with doses often being lower than those prescribed for younger adults^37^. In the absence of disease-specific regimens, standard dosing ranges are established based on the route of administration, ensuring consistency across age and weight groups. Renal adjustment dosing is particularly crucial for elderly patients with impaired creatinine clearance (CrCl), as it helps ensure safe antibiotic use by adjusting the dosage based on CrCl estimations^38^. This is essential, as renal function development in pediatric patients is often not fully considered in dosage adjustments, yet CrCl monitoring in all age groups ensures the safe and effective use of antibiotics^39^.

DOSAGE uses information about each patient to correctly figure out the right antibiotic dose for disease-specific, standard (unspecified disease), and renal adjustment regimens. This structure of individual dosage and administration recommendations provides methods for personalised antibiotic mediation.

#### Consideration of Risk Factors

Adverse effects are significantly increased in patients with underlying health conditions caused by antibiotics. The use of antibiotics needs special consideration of various contradictions to avoid unfavorable outcomes and ensure patient safety. Also, the use of antibiotics during pregnancy is very harmful to the health of the fetus, including the mother^40^. FDA categorized pregnancy risks into five classes (A, B, C, D, and X) to inform decisions about medication use during pregnancy by defining the potential dangers of a drug. Therefore, DOSAGE has integrated each antibiotic’s pregnancy risk category to ensure medication and patient safety.

### PHASE 2: Protocol Define for DOSAGE

After the requirement analysis, DOSAGE incorporates four essential elements: controlling standard dosage for known or unknown disorders, modifying dosages for renal function, taking pregnancy risk categories into account, and handling hypersensitivity issues. The physician informaticist (MZ) and chemist (TF) created the data model through an iterative process of discussion. In contrast, the informaticists (FA) conducted research to determine the necessary information for the features.

#### Data Model Development

**Fig. 1:**
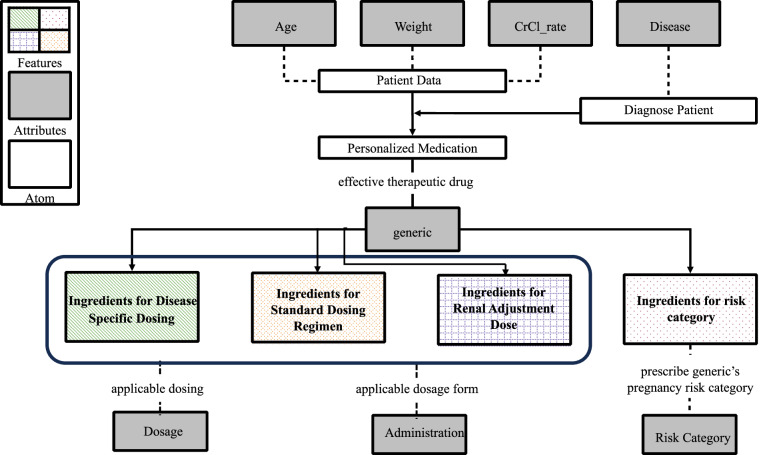
Data model flow according to define protocol of DOSAGE.

Figure 1 illustrates how DOSAGE’s data model ensures personalized antibiotic distribution by integrating patient-specific risk factors, such as age, weight, renal function, and pregnancy status. The variation in drug dosages for generic pharmaceuticals is primarily determined by the patient’s age, with weight often being an inconsistent factor in prescribing practices. The inclusion of a standard dosing regimen for each generic is crucial, as it provides a consistent baseline across the same age and weight groups, ensuring safe and reliable prescribing. While disease-specific regimens are tailored to particular conditions, the standard regimen serves as a universal framework, ensuring that patients without a defined disease condition still receive an appropriate dose. Additionally, kidney function, particularly the CrCl rate, significantly influences medication choices, especially in elderly patients. This consideration is crucial as renal adjustments are necessary to prevent drug toxicity. Furthermore, the method of administration may vary based on the patient’s age, medical condition, and pregnancy risk category. The design of the data model, as shown in Fig. 1, reflects these interdependencies by assigning unique identifiers to key data elements, including features, atoms, and attributes. The elements are interconnected via feature-based attributes, with atoms serving as the core building blocks of DOSAGE, representing the most basic and indivisible pieces of information inside the data model that offers a comprehensive framework for guiding antibiotic dosing decisions.

### PHASE 3: Data Collection

The data collection phase began with a curated list of 97 generic drug names, which served as the baseline dataset for subsequent analyses. This phase involved a structured approach to comprehensive pharmacological data collection through systematic steps.

#### Data Extraction and Extent Determination

**Fig. 2:**
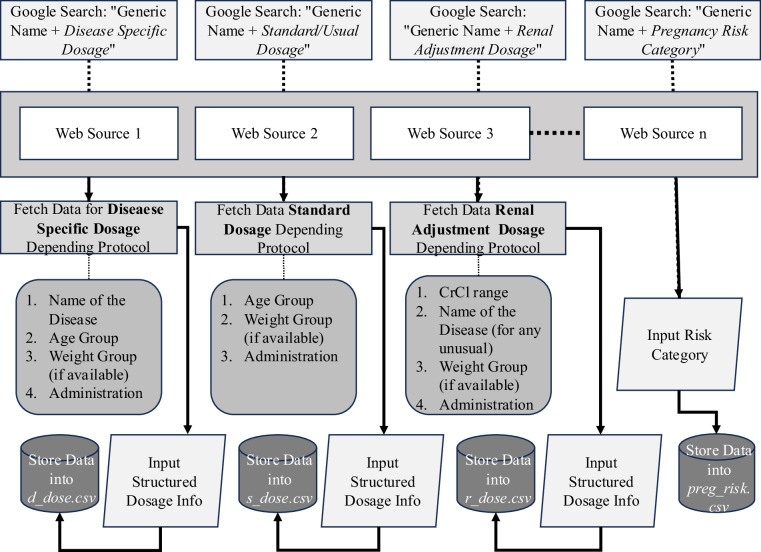
Protocol-wise manual data collection for antibiotics Medication.

Targeted Google searches were conducted for each generic drug to obtain detailed information on the four features. Coverage areas are defined for each feature depending on the criteria representing the DOSAGE features, as shown in Figure 2. Usual doses represent standard dosing regimens for each drug, whereas renal adjustment doses were created by modifying the evidence-based usual dose for patients depending on CrCl. Medications for which standard dose information was not available in literature-based web sources, we gathered medication information based on the expertise of medical professionals Table 1).

**Table 1:** List of sources and their origins used for data collection.

Name of Source	Origin
Drugs.com^18^	United States
MIMS^19^	Malaysia
Mayo Clinic^20^	United States
Medscape^21^	United States
Medical Dialogue^22^	India
MedEx^23^	Bangladesh
RxList^24^	United States

#### Preprocessing to Structure Dataset

Data preprocessing involved standardizing the raw data to create dataset. This was primarily due to the fluctuation of units and the need for range-based recommendations of the necessary attributes (shown in Table 2) from online sources. Following data collection, our data informatics (FW) divided key attributes such as age, weight, and dosage into multiple sub-attributes as shown in Table 2. This was done due to its potential advantage in developing automated techniques for medication correction and in collecting crucial information for verifying the correctness of prescription medications.

**Table 2:** Data description of DOSAGE.

Attributes	Columns	DOSAGE Table	Data Type	Description
Diagnosis	disease	*d_dose, r_dose*	String	Name of the disease for which the dosage is being adjusted
Age	min_age_d	d_dose, s_dose	Integer	Minimum range for the age group which is in days
	max_age_d			Maximum range for the age group which is in days
	min_age_m			Minimum range for the age group which is in in months
	max_age_m			Maximum range for the age group which is in in months
	min_age_y			Minimum range for the age group which is in in years
	max_age_y			Maximum range for the age group which is in in years
Weight	min_weight	*s_dose, d_dose, r_dose*	Numerical	Minimum weight in kg for the weight group
	max_weight			Maximum weight in kg for the weight group
CrCl rate	min_crcl	*r_dose*	Integer	Minimum CrCl rate for medication
	max_crcl			Maximum CrCl rate for medication
Dose	min_dd_mg	*d_dose, s_dose*	Numerical	Minimum daily dose in mg
	min_dw_mg			Minimum daily dose in mg according to weight
	min_dd_iu			Minimum daily dose in iu
	min_dw_iu			Minimum daily dose in iu according to weight
	lim_mg			Limit for DW type dosing in mg which should not exceed
	lim_iu			Limit for DW type dosing in iu which should not exceed
	max_dw_mg	*d_dose, s_dose, r_dose*		Maximum daily dose in mg according to weight
	max_dd_iu			Maximum daily dose in iu
	max_dw_iu			Maximum daily dose in iu according to weight
	max_dd_mg			Maximum daily dose in mg
Administration	route	*d_dose, s_dose, r_dose*	Categorical	The route of administration for medication
Remark	flag	*r_dose*	Categorical	Treatment recommendation remark for medication
Risk Factor	r_category	*preg_risk*	Categorical	Classification of generic based risk during pregnancy

### PHASE 4: Source Identification

Different online sites providing drug-related knowledge based on recognized literature served as sources of data for this study. In order to identify the sources for data informatics, there is a need for a thorough selection process. This criterion usually places emphasis on outpatient criteria in primary healthcare settings. Other key features to consider are those relevant to the dataset and should provide evidence-based information from the source. Thus, only those sources that satisfy all these criteria are chosen for the collection of data.

A team of specialists, including a physician (MZ) and a pharmacist (TF), conducts an additional level of verification once they have identified sources. This specialized examination evaluates not only the truthfulness, up-to-date, and applicability of information, but also its openness and availability, to determine the credibility of a source. Through this examination 7 sources were selected (showed in Table 1). The team also ensures that their sources are current and relevant, aligning with the standard practices in the medical field.

## Data Records

The dataset is available at Figshare^41^. Four interrelated tables named *d_dose*, *s_dose*, *r_dose*, and *preg_risk* were systematically structured there with generic serving as the unique identifier across all tables, collectively including nearly 4,000 clinically validated antibiotic medication combinations. The *d_dose* table provides precisely tailored disease-specific antibiotic dosing regimens for each patient groups. Wherease, same patient group without specifying any disease are provided in the *s_dose* table. Furthermore, the *r_dose* table facilitates dosage recommendations based on impaired renal function, measured by CrCl less than 90. Additionally, The category of pregnancy risk as a risk factor for antibiotics medication is classified in the *preg_risk* table, which supports safer prescribing practices.

### Patient’s Clinical Data

The patient data in this dataset is structured around key factors such as age, weight, and CrCl rate, which influence the appropriate antibiotic dosing. Some generics span multiple age groups due to varying disease treatment requirements. To address this, the age ranges were systematically organized during preprocessing, defining both the minimum and maximum age limits for each group. For example, antibiotics covering both 1 month older and 6 months older age groups would categorize the first as 1 to 5 months, with the next starting at 6 months. These ranges were further refined using relevant literature, where newborns are defined as under 28 days, and adolescence spans from 12 to 17 years. Moreover, Adult dosing generally starts at 12 years when no specific adolescent dosing is provided, with pediatric dosing continuing until 12 years. However, for geriatrics, the dosing aligns with adult levels, typically starting from 60 years. In terms of weight-based dosing, although rarely specified, weight limits are included where applicable. For instance, some disease-specific dosages apply to children aged 1 month to 11 years with a weight limit of 45 kg, while others, for the same or different diseases, may have no weight limit. When no weight specification is found in the literature, the field remains unfilled to maintain precision and adherence to medical standards. Similarly, for renal adjustment dosing based on CrCl, certain ranges may be underrepresented. For example, when a generic specifies a 1000 mg dose for patients with CrCl below 30 ml/min, higher CrCl levels are not addressed. Additionally, generics that lack specific disease indications or clear weight and administration guidelines are flagged as not recommended, ensuring the consistent application of safe and effective dosing practices.

### Prescribed Medication Data

To ensure accuracy and therapeutic relevance, DOSAGE’s primary goal is to personalize dosage regimens to match the unique needs of each patient cohort. This was accomplished by creating a comprehensive disease-specific dosing regimen in the *d_dose* table that accounts for 231 unique diseases, various age and weight groups, and three types of administration routes—oral (PO), intramuscular (IM), and intravenous (IV)—as well as generic medication combinations. Out of consideration for situations where no disease-specific regimen is justified, a standard dosage regimen is likewise comprised for every generic in *s_dose* table. Renal dose adjustments for antibiotics are typically based on CrCl levels, with most antibiotics following a standardized dosing approach regardless of disease. However, some conditions such as nosocomial pneumonia (for Piperacillin/Tambactam^42^) requiring higher doses or shorter intervals and pneumocystis pneumonia (for Trimethoprim/Sulfamethoxazole^43^) that require tailored reductions in severe renal impairment require disease-specific modifications due to unique pharmacokinetic demands. To ensure precise and comprehensive dosing, we incorporated a disease-specific renal dosing attribute in the *r_dose* table, prioritizing the highest allowable dose within each CrCl group while also considering weight range and administration type. Standard and disease-specific dosing regimens, on the other hand, include both minimum and maximum dose ranges. In order to provide the most precise dosage advice for each route, every regimen takes into account the change in dosage owing to the administration route.

Usually, the literature gives the minimum and maximum dose ranges for many combinations of generic, but occasionally it just says what dose is required. This issue was addressed by establishing the required dose as the minimum and adding an extra dose, considering the required dose as the maximum^44^. As indicated in Table 2, we discovered two distinct kinds of dosage types: dose per weight (dw), which varies according to the patient’s weight, and direct dose (dd), which is the dose directly defined without considering the patient’s weight. Though the dosage is correct in terms of the minimum and maximum range, the literature suggests that the daily limit cannot exceed a fixed limit, especially in the case of dw. Therefore, we have integrated these features into all dosage tables using the terms max_limit_mg and max_limit_iu because, doses might be prescribed in two different units (e.g., milligram (mg) and international unit (iu)) specific to generics. However, we must emphasise that the literature does not provide this data for all of the dw-mentioned dose recommendations, which makes it null at that sometime. Every dose suggestion in DOSAGE is based on clinical standards and takes into account the complete range of patient demands because these considerations are incorporated.

### Data for Medication Risk factors

The risk factors vary for each generic, remain consistent across all combinations of clinical and medication data outlined in *preg* table. According to the established protocol, DOSAGE takes into account the pregnancy risk factors for antibiotic medication. We found total 50 antibiotics fall into category B, which essentially means there is no risk in a human study, while 14 generics fall into category D, which has an effect on taking during pregnancy. While no generics are available in Category A or Category X, over 30 generics fall into Category C, which basically defines a risk that cannot be ruled out.

## Technical Validation

DOSAGE was developed using established data management practices, with validation conducted by a team of clinicians and data specialists. Data integrity, consistency, and clinical adherence were rigorously assessed through automated checks and manual reviews. The process ensured accurate demographic categorization, dosing regimens, and alignment with clinical protocols, guaranteeing DOSAGE’s readiness for safe and effective clinical use.

Verifying the correctness and homogeneity of age and weight categories across all dosing plans is the goal of the demographic range consistency check. The validation procedure ensures that each demographic category is properly represented by checking for gaps or inconsistencies between adjacent categories, specifically for age ranges. Incorporating all age categories into DOSAGE is ensured in this stage, as are all relevant patient populations. Similarly, when it comes to weight criteria for appropriate dosage, the validation process makes sure that the weight ranges are consistent and thorough. The check ensures that for every weight limit, the data for lower and higher weights are also provided. This method verifies that all patient demographics have been captured in DOSAGE, which helps to maintain dosage regimen uniformity and ensures that it meets clinical standards.

Compliance with clinical protocols is ensured by a rigorous two-stage validation process that incorporates collaborative corrections and independent evaluations. Medical professionals first carefully examine all standard, disease-specific, and renal adjustment dosage schedules to ensure that they follow established clinical guidelines. This check is essential for finding any problems or deviations from clinical standards, especially when it comes to age, weight, renal function, and pregnancy risk categories. Once anomalies are found, healthcare practitioners manually fix them to ensure that dosing regimens are entirely aligned with clinical standards. A different validation team checks these changes on their own as a part of the second step of the process. After making any necessary edits, this group double-checks the updated data to make sure it still follows all clinical guidelines. This two-step procedure conducts a comprehensive and independent verification to maintain the clinical integrity of data.

The completeness check ensures that the DOSAGE is complete and contains all relevant data points. This stage involves checking each table of DOSAGE to make sure that important information has been filled in accurately, which includes attributes like dosage ranges, patient data classifications, and pregnancy risk categories. The informatics (FW) is resolved when any data is missing or incomplete. This procedure is essential for clinical decision-making since it guarantees that no data will be missing and provides the foundation for a complete dataset. This validation process confirms dataset is reliable and suitable for clinical use by checking that all necessary fields are filled out.

## Usage Notes

DOSAGE is a comprehensive dataset designed to enhance automated systems in evaluating the appropriateness of antibiotic prescriptions by integrating patient-specific data, including medical conditions, renal function, and pregnancy status. It ensures compliance with medical standards by maintaining dosing regimens within safe limits, thereby promoting patient safety and adherence to best practices. DOSAGE includes a wide range of dosing regimens for all antibiotic medications, making it universally applicable and a valuable resource for researchers and clinicians alike^45,46^. Researchers can integrate DOSAGE into clinical decision support systems or machine learning algorithms to refine dose precision and optimize therapeutic outcomes, particularly in combating antimicrobial resistance, a growing global health concern^45,47^. While large language models can interpret unstructured text and generate context-aware responses, they still require structured and validated references to ensure safe and accurate outputs. DOSAGE provides such a foundation by offering clinically grounded, patient-specific logic that can be used to refine, validate, or filter LLM-generated recommendations. Furthermore, when embedded into an electronic prescribing platform, DOSAGE can generate real-time alerts that notify clinicians of dose deviations, renal-adjustment or risk factor for pregnancy needs before a prescription is finalized. To operationalize this capability, a lightweight web application (http://www.dosage.iriic.uiu.ac.bd) has been developed that translates clinical inputs into validated dosing and administration guidance in real time. This interface supports both individual and batch prescription verification, enabling seamless integration into clinical and research workflows that rely on rule-based support system. The tables of dataset are formatted in CSV using Microsoft Excel, ensuring ease of access and integration into computational tools. Importantly, DOSAGE prioritizes privacy by excluding sensitive patient information and focusing on parameters such as age, weight, and creatinine clearance to recommend suitable medications. This approach aligns with global efforts to improve antibiotic use and mitigate resistance, as highlighted by databases like ACDB^45^ and tools like VAMPr^48^, which leverage structured data to predict antibiotic efficacy and resistance patterns. By providing a standardized and accessible resource, DOSAGE contributes to the development of data-driven strategies in antibiotic prescribing, ensuring safer and more effective patient care.

## Data Availability

Python was used for data filtering and analysis. Although no bespoke code was required for dataset curation, a demonstration notebook (dosage_demo.ipynb) with example scripts is provided in the repository^41^ to facilitate reproducible exploration and filtering.
